# Supplementary material for: Some new applications of the fractional integral and four-parameter Mittag-Leffler function
Source: PLoS One. 2025 Feb 3;20(2):e0317776. doi: 10.1371/journal.pone.0317776 (PMC11790175; doi:10.1371/journal.pone.0317776)
Supplement: Attachment — Submitted filename: Review Report Plos One.pdf [file pone.0317776.s001.pdf]

Review report:

Title : Some New Applications of the Fractional Integral and Four-Parameter Mittag-Leffler Function

Authors: Ahmad A Abubaker, Khaled Matarneh, Suha B. Al-Shaikh1, Mohammad Faisal Khan

In this article authors present innovative applications of the fractional integral of the four-parameter Mittag-Leffler function in the Geometric Function Theory. This study defines a new integral operator of order λ using the fractional integral and four-parameter Mittag-Leffler function. The authors employ differential subordination technique to derive geometric features satisfying univalence conditions for the newly defined fractional integral operator of Mittag-Leffler function. The paper demonstrates rigorous mathematical derivations and proofs, ensuring the correctness and reliability of the results. Using the concepts of remark 1.2, they proved that the newly defined operator is starlike, convex, and close-to-convex for positive and negative orders, respectively.

The manuscript is well-structured and clear, with precise mathematical formulations and thorough proofs. The abstract, discussion, and conclusion effectively summarize the research. However, the authors should revise the introduction section to clearly state the motivation behind this new work and explicitly address the gap between existing research and the new contributions presented. Additionally, the introduction should provide a clearer context by referencing relevant and recent articles related to their work.

1. Applications of a q-Differential Operator to a Class of Harmonic Mappings Defined by q-Mittag–Leffler Functions, Symmetry, 14, 1905 (2022), doi.org/10.3390/sym14091905.
2. Faber polynomial coefficients estimates for certain subclasses of q-Mittag-Leffler-Type analytic and bi-univalent functions, AIMS Mathematics, 7(2) (2021), 2512-2528.
3. Some Applications of Analytic Functions Associated with q-Fractional Operator, Mathematics, 11, (2023), 930.https://doi.org/10.3390/ math110409.

I would like to bring to your attention some errors and suggestions

1. In the abstract line 4: It should be “four-parameter Mittag-Leffler function ”
2. In the abstract, line 7: Replace “specified” with “proved”.
3. On page 9, line 23: Replace “specific instances “ with special cases”

4. On page 12, in the start of the line 5, remove the extra words “some new results”.
5. Check for typos error in the whole paper.
6. The conclusion section lacks clarity on future directions. Please revise and expand the conclusions to include specific future research directions

Decision: This paper by Abubaker et al. makes a substantial contribution to fractional calculus and Geometric Function Theory through differential subordination. The results are well formulated, new and easily understandable. **After the above mentioned changes, the article can be published in “Plos One”.**
