# Supplementary material for: Some new applications of the fractional integral and four-parameter Mittag-Leffler function
Source: PLoS One. 2025 Feb 3;20(2):e0317776. doi: 10.1371/journal.pone.0317776 (PMC11790175; doi:10.1371/journal.pone.0317776)
Supplement: Attachment — Submitted filename: Response to Reviewers.pdf [file pone.0317776.s002.pdf]

Reviewer≠1: Comments to the Author
REVIEWER's REPORT
On the paper PONE-D-24-38839
Some new applications of the fractional integral and four-parameter Mittag-Leffler function
by Ahmad A Abubaker, Khaled Matarneh, Suha B. Al-Shaikh, Mohammad Faisal Khan

The results are new, correct and detailed. The paper is original and doesn't contradict to ethical or policy issues, the question posed by authors is new and well defined, the methods used by authors are appropriate and well described, the data are sound and well controlled, the discussion and conclusions are well balanced, the title and abstract convey the obtained results, the writing is acceptable, the paper contains good scientific results.
The paper doesn't require a revision.
Taking the above into consideration, I recommend the paper for publication in PLOS ONE..
Reply:
Respected Professor
Thank you for your careful reading and remarks. We have made some small changes for the improvement of our paper in the revised version. All changes are highlighted in the revised version's PDF.

Reviewer≠2: Comments to the Author

In this paper, the authors obtained the theorems about the necessary and sufficient conditions for the operator of the four-parameter Mittag-Leffler function using fractional calculus. Then, the authors showed the fractional integral operator of the four-parameter must satisfy the conditions of starlikeness and convexity. Finally, the study of geometric functions theory, differential subordination, and superordination theories were given. The main results are interesting. Some specific comments are presented as follows.

1) Some definitions and lemmas should be given as the preliminaries section.

Reply to the Reviewer: Respected professor according to your suggestions we made a new sections name as preliminaries for some definitions and lemmas in the revised version.

2) The main motivations and contributions of this paper should be clarified in the introduction part.

   Reply to the Reviewer: we have added new paragraph in the revised version which clearly explain the main motivations and contributions of this paper. See the highlighted part of revised version.

3) Some comparisons should be given to show the advantage of this paper.

   Reply to the Reviewer : we have added new paragraph in the revised version which clearly explain the advantage of this paper of this paper. See the highlighted part of revised version.

4) Do you provide some examples to show the effectiveness of main results?

   Reply to the Reviewer: Respected professor, we have added one theoretical example in the revised version.

Reviewer≠3: Comments to the Author
The manuscript is well-structured and clear, with precise mathematical formulations and thorough proofs. The abstract, discussion, and conclusion effectively summarize the research. However, the authors should revise the introduction section to clearly state the motivation behind

this new work and explicitly address the gap between existing research and the new contributions presented. Additionally, the introduction should provide a clearer context by referencing relevant and recent articles related to their work.
1. Applications of a q-Differential Operator to a Class of Harmonic Mappings Defined by q-Mittag–Leffler Functions, Symmetry, 14, 1905 (2022), doi.org/10.3390/sym14091905.
2. Faber polynomial coefficients estimates for certain subclasses of q-Mittag-Leffler-Type analytic and bi-univalent functions, AIMS Mathematics, 7(2) (2021), 2512-2528.
3. Some Applications of Analytic Functions Associated with q-Fractional Operator, Mathematics, 11, (2023), 930.https://doi.org/10.3390/ math110409.
Reply to the Reviewer: Thanks for highlighting the related article. We added these articles in our manuscript. Researchers can extend this work by using the concepts of above mentions papers.

I would like to bring to your attention some errors and suggestions
1. In the abstract line 4: It should be “four-parameter Mittag-Leffler function ”
2. In the abstract, line 7: Replace “specified” with “proved”.
3. On page 9, line 23: Replace “specific instances “ with special cases”
4. On page 12, in the start of the line 5, remove the extra words “some new results”.
5. Check for typos error in the whole paper.
Reply to the Reviewer: We have incorporate the point 1-5, in the revised version.
6. The conclusion section lacks clarity on future directions. Please revise and expand the conclusions to include specific future research directions.
Decision: This paper by Abubaker et al. makes a substantial contribution to fractional calculus and Geometric Function Theory through differential subordination. The results are well formulated, new and easily understandable. After the above mentioned changes, the article can be published in “Plos One”.
Reply to the Reviewer: According to your suggestions, we have added future directions in the revised version, which are highlighted in revised version.

Reviewer≠4: Comments to the Author

The paper addresses a topic that provides very interesting results and is intensely investigated nowadays which concerns the embedding of fractional calculus aspects into geometric function theory. The introduction of new fractional operators is a research theme of interest at the moment and this is well evidenced in the text of the paper. The new integral operator introduced in this paper is valuable due to the properties proved in this paper which can open ways for future investigations by applying this operator in the theories of differential subordination and superordination and other fields that apply fractional operators. The motivation of the paper is clearly presented, all the necessary notions and previously established results are highlighted in the Introduction and all the references cited in the text are relevant. The paper is well organized and easy to follow. The proofs are correct and contain all the necessary details.

1. The paper needs small adjustments regarding the English language. Most important, the Abstract should be rephrased.
2. page 3: "Let H(U) represents" should be "Let H(U) represent" and the sentence should begin at a new paragraph. "...is the subclass of H(U) which is define as:" should be "...is the subclass of H(U) which is defined as:"

Reply to the Reviewer:
Respected Professor,

Thank you for your careful reading and remarks. We have made every effort to eliminate all typographical and language errors. Further, we have rephrased abstract according to your suggestion. All changes are highlighted in the revised version PDF.
